# A Greenhouse Gas and Soil Carbon Model for Estimating the Carbon Footprint of Livestock Production in Canada

**DOI:** 10.3390/ani2030437

**Published:** 2012-09-04

**Authors:** Xavier P.C. Vergé, James A. Dyer, Devon E. Worth, Ward N. Smith, Raymond L. Desjardins, Brian G. McConkey

**Affiliations:** 1 AAFC Consultant, Ottawa, ON, K2A 1G6, Canada; E-Mail: Xavier_vrg@yahoo.fr; 2 AAFC Consultant, Cambridge, ON, N3H 3Z9, Canada; E-Mail: jamesdyer@sympatico.ca; 3 Eastern Cereal and Oilseed Research Centre, Agriculture and Agri-Food Canada (AAFC), Ottawa, ON, K1A 0C6, Canada; E-Mails: Devon.Worth@agr.gc.ca (D.E.W.); ward.smith@agr.gc.ca (W.N.S.); 4 Semiarid Prairie Agricultural Research Centre, Agriculture and Agri-Food Canada (AAFC), Swift Current, SA, S9H 3X2, Canada; E-Mail: Brian.McConkey@agr.gc.ca

**Keywords:** greenhouse gas, soil carbon, carbon footprint, payback period, beef, pork

## Abstract

**Simple Summary:**

We developed a model to estimate the carbon footprint of Canadian livestock production. To include long term soil carbon storage and loss potential we introduced a payback period concept. The model was tested by reallocating 10% only of the protein production from a ruminant to a non ruminant source to minimize the risk of including rangeland or marginal lands. This displacement generated residual land which was found to play a major role in the potential mitigation of GHG emissions. The model will allow land use policies aimed at reducing the agricultural GHG emissions to be assessed.

**Abstract:**

To assess tradeoffs between environmental sustainability and changes in food production on agricultural land in Canada the Unified Livestock Industry and Crop Emissions Estimation System (ULICEES) was developed. It incorporates four livestock specific GHG assessments in a single model. To demonstrate the application of ULICEES, 10% of beef cattle protein production was assumed to be displaced with an equivalent amount of pork protein. Without accounting for the loss of soil carbon, this 10% shift reduced GHG emissions by 2.5 TgCO_2_e y^−1^. The payback period was defined as the number of years required for a GHG reduction to equal soil carbon lost from the associated land use shift. A payback period that is shorter than 40 years represents a net long term decrease in GHG emissions. Displacing beef cattle with hogs resulted in a surplus area of forage. When this residual land was left in ungrazed perennial forage, the payback periods were less than 4 years and when it was reseeded to annual crops, they were equal to or less than 40 years. They were generally greater than 40 years when this land was used to raise cattle. Agricultural GHG mitigation policies will inevitably involve a trade-off between production, land use and GHG emission reduction. ULICEES is a model that can objectively assess these trade-offs for Canadian agriculture.

## 1. Introduction

The growing global demand for food will compete with efforts to mitigate Greenhouse Gas (GHG) emissions and adapt to climate change [1,2]. With the expansion of high protein diet among many emerging economies, large land areas will be required for livestock feed production [3,4]. This land use will compete with crops for direct human consumption or biofuel feedstock [5,6]. There is also concern about the large amounts of enteric methane emitted by ruminant livestock [7], as well as the emissions of nitrous oxide and carbon dioxide from all types of livestock operations [8]. Important questions arise about which types of livestock satisfy the demand for protein most efficiently, make the best use of the land resource base and have the lowest carbon footprint. Therefore, to help the livestock industries cope with these pressures, an objective set of algorithms that can compare how various livestock types impact the environment and meet growing food demands will be needed. 

Sequestering atmospheric carbon dioxide (CO_2_) as soil carbon is a potential strategy for reducing GHG emissions [9]. About one third of the soil carbon stock was lost when virgin soils were first broken with the plow in Canada and this stock declined further under continued mechanized cultivation [10]. Better farming practices over the last twenty years, however, have reversed this trend. Canada’s agricultural soils, which were estimated to have been a small CO_2_ source in 1991, were a small sink by 2001 [11]. But there are tradeoff effects with regard to the extent to which all of these practices are applied and which crops are grown. For example, increases in nitrous oxide (N_2_O) and methane (CH_4_) emissions from livestock and crop productions have been offset by carbon sequestration in soils [11]. Forage crops enhance soil organic matter, but feeding those crops to livestock increase CH_4_ emissions [12]. 

The main objective of this paper was to present a dynamic, quantitative model for estimating GHG emissions and determining the carbon footprint of Canadian livestock industries. To determine the long term impact of changes in livestock populations on the carbon footprint of Canadian farms, this model integrates the changes in soil carbon associated with shifts among livestock populations with their annual GHG emission budgets. The second objective was to demonstrate the linkages among GHG emission calculations that are livestock type specific and their relationship with soil carbon. To achieve the second objective four scenarios for livestock industry interactions (described below) were assumed.

## 2. Experimental Section

### 2.1. Development of a Livestock GHG Model

#### 2.1.1. Background

Commodity assessments have been previously completed for the Canadian dairy, beef pork and poultry industries [13,14,15,16]. However, these assessments did not consider inter-commodity interactions. Satisfying livestock diet requirements can lead to competition for feed grain, especially under intensive animal production. Since livestock industries must share arable land with food crops, oilseeds [6] and biofuel feedstock crops [5,17], livestock industries can no longer be treated as separate closed systems. The Unified Livestock Industry and Crop Emissions Estimation System (ULICEES) was created by assembling the four sets of livestock GHG computations in one model. ULICEES also takes the changes in the soil carbon stock into account. Although ULICEES is applicable to any agricultural census year, in this analysis it was applied to 2001, the most recent year for which the livestock diet survey data was available [18]. 

**Figure 1:**
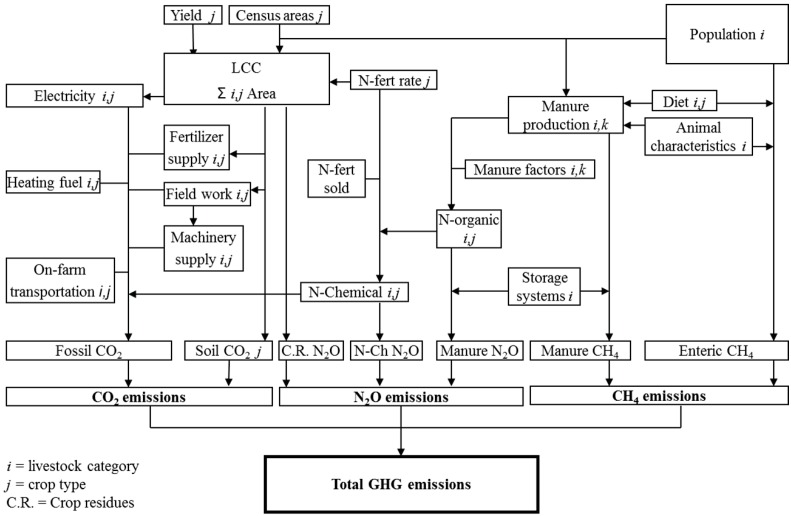
Chart of the generalized computational flow of the Greenhouse Gas (GHG) emission budgets of the four major Canadian livestock industries.

Figure 1 presents the generalized computational flow of the set of calculations for quantifying the GHG emission budgets for Canadian livestock production. Only the functions common among all four livestock industries are illustrated. The yields, areas, and fertilizer application rates for each crop and population for each livestock type are shown as computational inputs. The resulting GHG emission categories and totals are shown at the bottom of the chart. Although ULICEES addresses the question of how to measure the carbon footprint of all food of animal origin in Canada [8], this chart does not show interactions among commodities and it only considers changes in soil CO_2_ emissions under a land use change. All of the GHGs associated with animal housing [19] were also taken into account in ULICEES. 

#### 2.1.2. Crop Complex

The scope of assessment adopted by Vergé *et al.* [14] included the associated complex of crops that supported each commodity production system. The carbon footprint of animal based production cannot be effectively quantified without first determining the GHG emissions from growing the feed grains and the forage they consume. That land base, defined as the livestock crop complex (LCC), was the result of integrating the diets of all the age-gender categories in each livestock type over the whole population. Each crop component of each diet was divided by the average yield of that crop to estimate the land required to grow the crop. 

Specific crop complexes, DCC, BCC, PCC and ACC, were defined for the dairy, beef, pork and poultry (avian) industries, respectively, in Canada [4,13,14,15,16]. The LCC was a critical step in integrating livestock commodities in ULICEES. Livestock ration records obtained from Statistics Canada [18] were combined with animal population data from agriculture census records [20,21] to calculate the LCC of each livestock industry. By defining the area needed to feed all on-farm animals, the LCC concept sets limits on the livestock production system. It recognizes that the GHG emissions attributed to livestock do not stop at the feedlot or the animals. Even if farmers buy all their livestock feed, the GHGs emitted from the land on which those crops were grown were still attributed to those livestock. 

#### 2.1.3. The GHG Emissions and Sources

Like the four previous commodity specific GHG emission assessments, ULICEES accounted for CH_4_, N_2_O and fossil CO_2_. Since these emission calculations were described in detail in the previous assessments [4,14,15,16], only a general overview of these calculations is described here. Enteric methane emissions from ruminants were based on the Intergovernmental Panel on Climate Change (IPCC) Tier 2 methodology [22], adapted for Canadian conditions by Vergé *et al.* [23]. These emission estimates also accounted for a small but measurable amount of enteric methane from hogs [24]. The default IPCC tier 1 emission factors were used and corrected based on the animal weight as presented by Vergé *et al.* [15]. Methane emissions from manure were calculated using the IPCC Tier 2 methodology [22] for all animal types. Methane emission factors for each age gender category were then multiplied by their respective populations.

Based on the IPCC tier 1 methodology the total amount of nitrogen applied in each LCC was used to define the N_2_O emissions from each livestock production system [22,25]. The N_2_O emission factors adapted to Canadian conditions came from Rochette *et al.* [26]. Different computation pathways were required depending on whether the nitrogen source was organic or chemical. The annual amount of organic nitrogen applied was based on the quantity of manure produced by each livestock population and the nitrogen content of each type of manure. The amount of chemical nitrogen fertilizer applied was obtained by subtracting the organic nitrogen from the total required nitrogen. Crop specific nitrogen fertilizer application recommendations [27] were integrated over the respective LCC crop areas to derive the total nitrogen in the LCC. The amount of chemical nitrogen was adjusted to the amount of fertilizer actually sold in each region [28]. This adjustment factor was calculated by comparing the total recommended amount of nitrogen calculated and assumed to be applied within each province to the total nitrogen fertilizer purchased in the same province [4,14,15,16]. 

Although smaller in magnitude than agricultural methane and nitrous oxide emissions, farm energy is an essential part of the sector’s GHG emissions budget [29,30]. A combination of farm statistics and agricultural engineering coefficients were used to estimate the fossil CO_2_ emissions from the fossil fuel for farm fieldwork [31,32]. The energy and fossil CO_2_ emissions associated with on farm transport, farm use of electricity and heating fuel and the indirect fossil energy to manufacture and transport farm machinery and chemical fertilizer to the farm were also included [30,33,34]. 

#### 2.1.4. The Soil Carbon Stock

In previous commodity specific applications of the LCC methodology, CO_2_ emissions from soil carbon were not considered because each livestock system was treated in isolation with little change in overall land use or management. ULICEES treats soil carbon as an exhaustible storage term, including changes in soil organic carbon (SOC) between different land-use management systems in a similar manner as IPCC GHG accounting methodology [22], whereby a land-use/management system that is in equilibrium is converted to a new land-use/management system and is assumed to reach a new equilibrium within a time frame. Some forage fields that are in rotation with annual crops may not be at equilibrium. For these cases, additional atmospheric CO_2_ which would have been sequestered if those forage areas in rotation had been given time to come to equilibrium, along with the sequestered soil carbon. Including that lost sequestration potential would, therefore, result in the same total loss of sequestered CO_2_ as lost from the soil under continuous perennial forage.

ULICEES assumes a new SOC equilibrium after 40 years. We assumed that after 40 years SOC stabilizes to a new soil organic carbon level where it no longer makes an appreciable contribution to the annual GHG emissions budget. As well, 40 years from the present, 2050, is approximately the time when GHG levels in the atmosphere were projected at the Nairobi Climate Change Summit (COP 12) to double compared to pre-industrial atmospheric levels [35]. For each livestock production system, yearly GHG emissions of CH_4_, N_2_O and fossil CO_2_ continue indefinitely as long as that production system operates. Carbon flow is, however, not permanent or linear from a long term carbon balance perspective [11].

### 2.2. The Payback Period

Based on the difference in GHG emissions from beef and pork, a period can be calculated that is required to accumulate a multi-year quantity of GHG emissions that equal the difference in carbon sequestration between the two land uses. The time required to compensate for an amount of soil carbon that is lost as a result of a land change is defined as the payback period. This payback period relates the loss of soil carbon to the annual GHG emissions. A precedent has been set for the payback period approach in life cycle assessments of biofuels [36,37,38,39]. For example, if a tropical forest is cut down to grow palm oil for biodiesel feedstock, it would take many decades before the annual offset of fossil CO_2_ emissions from the biodiesel equals that loss in tropical soil carbon. The beef to pork conversion can be treated in the same way. The change in crop areas for the beef to pork redistribution would come from the forage area that supported the displaced population of beef which would then be converted to feed grain for additional hogs. 

Since the complete decay curve for soil carbon between two steady states is exponential, its slope approaches zero asymptotically near the equilibrium. Hence, the decay period must be defined in terms of a tolerable amount of residual soil carbon. In this analysis, the decay period was set at 40 years which accounts for about 60% of the carbon stock [40]. The remaining 40% would be lost over the next 60 years at an average annual rate that is less than half of the average soil carbon decay rate over the first 40 years. The integrated annual GHG emissions over the payback period can be compared to the change in soil carbon stock over that period. A payback period that is appreciably shorter than the 40 year decay period represents a net gain in GHG mitigation potential. 

### 2.3. The Beef to Pork Redistribution

To demonstrate the inter-commodity interactions, a potential expansion of the pork industry was assumed which would displace some of the beef industry in Canada. Whereas beef cattle in Canada are raised mainly on roughages supplemented by grain, hogs are completely dependent on annual crops. Therefore, additional feed grain area would be required for the expanded hog population. This livestock redistribution illustrates the tradeoff between the reduction of enteric methane emissions and the soil carbon loss during the replacement of perennial forage cover with annual crops. Related changes in the LCC will include more fossil fuel use for farm field operations and an increase in N_2_O emissions due to higher nitrogen fertilizer requirements. 

The quantitative basis of the beef to pork redistribution test was to avoid any loss of protein supply. Hence, the increase in pork production must supply the same amount of protein as was lost from beef production. The two quantities of protein were calculated using the protein to live weight conversion factors from Dyer *et al.* [41]. The beef and pork systems have different intensities based on protein production [41], These differences in GHG emission intensities can also be seen in beef and pork comparisons based on live weight production [4,42,43,44]. However, it was not the objective of this paper to compare productivities of these industries, but to ensure that the loss in food production from the land use change would be minimized.

Because the BCC can include land that is typically only suitable for growing perennial forages [45], only a portion of the BCC can be reallocated to grow annual crops in the PCC to feed hogs. As recognized by Basarab *et al.* [44], the chance of some of that redistributed land being of too low quality to grow the additional feed grains for hogs had to be minimized. By transferring only 10% of the beef based protein production potential to pork production, only the best portion of the BCC was allowed to be involved in the redistribution of land in both eastern and western Canada. The increase in pork production as a percentage of the total annual supply of protein from pork must be larger than 10% where the supply of protein from pork is less than the total amount of protein from beef. It must be less than 10% where the pork protein supply exceeds the supply of protein from beef. Whereas the deflation factor for beef was 90% for both eastern and western Canada, the corresponding inflation factors for pork were different between the two Canadian regions (Table 1). 

**Table 1:** Weights of protein before and after redistribution from beef to pork production and the beef deflation and pork inflation factors.

	Initial	Reallocated	Remaining	After/before
		kt, protein		factors (%)
		Beef		for deflation
East	36.9	3.7	33.2	90
West	218.8	21.9	197.0	90
		Pork		for inflation
East	157.7	3.7	161.4	102
West	123.5	21.9	145.4	118

The beef to pork redistribution involves three crop area changes: area going into feed grains for pork (A*p*); the forage area that was supporting displaced beef (A*f*); and the feed grain area that was supporting displaced beef (A*g*). Some of the area required to grow feed grain for the hogs had to be taken from land that had been growing forage. The conversion from forage to annual grains determined the area (∆*_c_*A) in which the changes in soil carbon storage caused solely by the expansion of pork production take place. The initial land displacement was computed as:

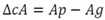
(1)

Not all of the land in forage was needed to expand the feed grain crop to support more hogs. Being a more extensive system, there will be land left over from the beef industry as the required perennial forage is reduced. The GHG emissions of the residual forage area (∆*_r_*A) could undergo a further change because it is no longer needed by the displaced beef cattle. The residual area from the initial land displacement was computed as:

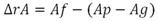
(2)

### 2.4. Residual Land Redistribution Scenarios

It is difficult to predict with any certainty the most likely land use for ∆*_r_*A from the beef to pork redistribution. Because of this uncertainty, four scenarios for the residual forage area were examined. These scenarios result in a range of impacts on the total carbon footprint of the redistribution from the perspective of the land use and crop choices for ∆*_r_*A.

- Scenario 1 assumes that ∆*_r_*A will remain under perennial forage cover.
- Scenario 2 assumes that ∆*_r_*A will all be seeded to annuals, such as for food (bread quality wheat or cooking oil) or feedstock for grain ethanol or canola biodiesel.
- Scenario 3 assumes that ∆*_r_*A will be returned to beef production with the same overall herd structure and, hence, the same overall GHG emission rates per ha.
- Scenario 4 assumes that ∆*_r_*A will be returned to beef production with a mainly grass fed population whose diet included more forage and less grain than the beef cattle in Scenario 3.

Scenario 1 can be considered a baseline for the other three scenarios. Scenarios 2, 3 and 4 increase the carbon footprint of the beef to pork redistribution and had to be subtracted from the initial GHG emission savings. Area based GHG emission rates for edible pulses and cereals defined by Dyer *et al.* [45] were used in Scenario 2 to account for growing annual crops on ∆*_r_*A. In Scenarios 3 and 4, the emissions to be subtracted from the initial GHG emission savings come from repopulating ∆*_r_*A with beef cattle. For Scenario 3, the emissions to be subtracted were based on the whole beef population. For Scenario 4, the emissions to be subtracted were based on just those parts of the beef population that were not being fattened for slaughter with feed grain supplements. 

## 3. Results and Discussion

### 3.1. Results

#### 3.1.1. Canadian Livestock GHG and Land Use Inventory

**Figure 2:**
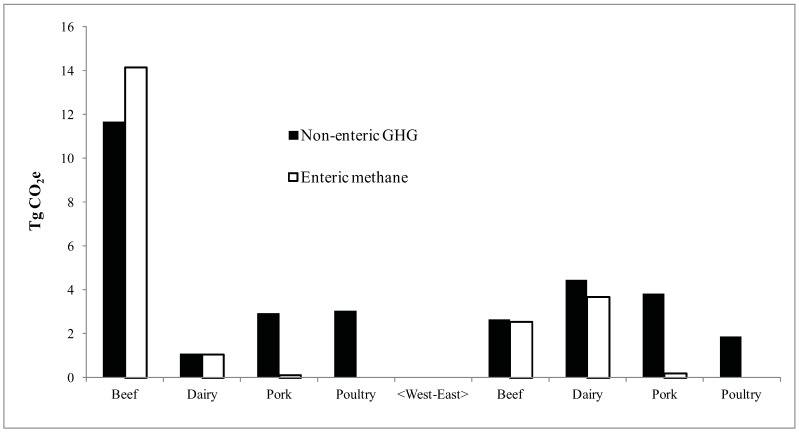
Greenhouse gas (GHG) emissions from four types of livestock in eastern and western Canada separated into enteric and non-enteric sources (land use and manure storage systems) in 2001.

A summary of GHG emissions from the four livestock industries for eastern (Atlantic Provinces, Québec, Ontario) and western (Manitoba, Saskatchewan, Alberta, British Columbia) Canada is shown in Figure 2. These quantities reflect the respective sizes of the four industries as much as differences in GHG emission types. Since changes in soil carbon relate to interactions among livestock populations, GHG emissions were grouped in a way that most closely relates to ruminant and non-ruminant livestock systems. Hence, the GHG emissions in Figure 2 are distinguished as either enteric or non-enteric. Non-enteric GHG emissions include manure methane, N_2_O from both the soil and stored manure, and fossil CO_2_. The main sources of the non-enteric GHGs are the annual crops that supply the feed grains for non-ruminants (hogs and poultry), and the grain component of cattle diets. Canadian livestock accounted for 53 TgCO_2_e in 2001 with 22 TgCO_2_e coming from enteric methane. The Canadian beef industry emitted 31 TgCO_2_e. Western beef accounted for 26 TgCO_2_e, 14 of which were enteric methane. Dairy and pork production accounted for 10 and 7 TgCO_2_e, respectively. At 5 TgCO_2_e, poultry was the lowest source of GHG from the livestock industry in Canada. 

**Figure 3:**
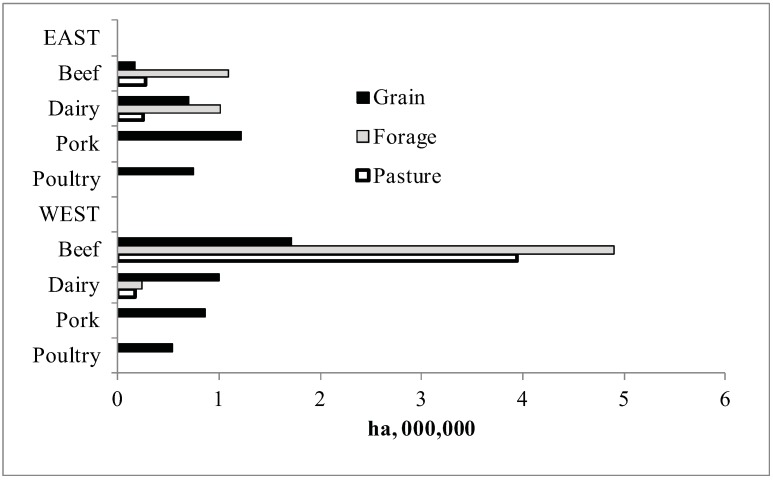
Areas in the livestock crop complex in each of four types of livestock in eastern and western Canada in 2001 grouped by three general land use classes.

Figure 3 shows the areas in each of these four crop complexes and groups land use according to those cultivated for grain, harvested forage, or improved pasture. The BCC and DCC include all three classes of land (grain, forage and pasture), but the only land in the PCC and ACC is land that can produce grains and pulses (annuals). Since soil carbon is generally higher under perennial forage than under annual crops [10], a shift from ruminant to non-ruminant livestock production would reduce soil carbon stock. Silage corn, although an annual crop, was grouped with the forages in Figure 3. Pasture represents an appreciable land use only in the western beef industry. It was assumed in this study that most of that land would be unsuitable, or at least the last land selected, for reseeding to grow annual feed grains or harvested field crops. Hence, that land would most likely continue to be under permanent (perennial) cover under the 10% livestock redistribution scenarios examined in this paper. 

#### 3.1.2. Impacts of Beef to Pork Redistribution on Crop Lands and GHG Emissions

In Table 1, the pork inflation factor is closer to 100% than the beef deflation factor (90%) in the east because the total supply of pork protein is much greater than the total supply of protein from beef in that region. The opposite is true in the west because the total supply of beef protein is higher than the total pork protein supply in that region. The post-redistribution areas in Table 2 reflect the percentages shown in Table 1, whereby the proportional increase in land resources allocated to pork production is smaller than the proportional decrease in areas supporting beef in the east, but is larger in the west. As expected, the new areas to produce feed grains for the expanded hog population exceeded the reduction in areas that had produced feed grains and silage corn for beef in both regions. Table 2 also shows that silage corn was the dominant annual crop for beef feed in the east, but was not important in the west. Even though there is 3.4 times as much land in these two industries in the west (Table 2), the total protein supplied by beef and pork from the west only exceeds the east by 80% (Table 1).

**Table 2:** Crop areas that support the beef and pork industries before and after redistribution of land from beef to pork production.

		Beef		Hogs
	Feed grain	Silage corn	Harvested perennials	Feed grain
		Before redistribution (ha.10^3^)		
East	117	98	1,000	1,219
West	1,818	42	4,944	1,641
Canada	1,994	140	5,944	2,860
		After redistribution (ha.10^3^)		
East	159	88	900	1,247
West	1,636	38	4,449	1,932
Canada	1,795	126	5,350	3,179

The new area in annuals in Table 3 (first column, ∆*_c_*A) does not affect GHG emissions among the four scenarios because the emissions are already accounted for in the area of expanded pork. The areas in the second column, ∆*_c_*A + ∆*_r_*A, signify the initial reduction of the beef population, before reallocating ∆*_c_*A back to the pork industry. The third column, ∆*_r_*A, takes the areas required for the expanded pork production system shown in Column 1 into account. Column 3 is the result of the differences in areas in Table 2 for changes in both livestock populations. Because of the stronger role of silage corn in the eastern beef diet, the encroachment into perennial forage land area for grain production for hogs (Column 2) was much lower in the east. The portions of ∆*_r_*A that must be used to grow feed grain for the repopulated beef in the third and fourth scenarios are shown in the last two columns of Table 3. The areas for growing additional feed grain are about a third higher for Scenario 3 compared to Scenario 4. These relatively small portions of ∆*_r_*A reflect the lesser role of grains in the diet of the repopulated beef cattle. 

**Table 3:** Changes in area in the beef crop complex as a result of reducing beef production and expanding pork production, and after repopulating the residual forage area (∆*_r_*A) with beef cattle.

	New area in	Area remaining from		Annuals to support new beef	
	annuals (∆_c_A)	harvested perennial forage		Mix of forage	Mainly
	beef to pork	Initial ^1^	Residual ^2^	and grain	grass-fed
Regions	ha,000				
East	1.1	100.0	99.0	21.3	16.6
West	104.7	494.4	389.7	106.5	77.1
Canada	105.8	594.4	488.6	129.1	93.4

^1^ area freed after initial reduction in the beef cattle displaced by hogs (∆cA + ∆rA) ^2^ perennial area remaining after re-seeding to annuals to feed more hogs (∆rA)

The GHG emission budgets for beef and pork production prior to the four scenarios are presented in Table 4. The first group of four rows illustrates the basic livestock-specific GHG simulations generated by ULICEES. The western beef industry is by far the largest source of GHG emissions, and methane from western beef is the largest term in the combined GHG budget of these two industries. There was much less east-west difference in emissions in the pork industry, but both were lower than the eastern beef industry emissions. Fossil CO_2_ was the lowest GHG emission from both industries while CH_4_ was the highest. The second and third groups of two rows represent GHG deducted from beef and added to pork production, respectively. The remaining data in Table 4 show the net potential savings or reductions in annual GHG emissions as a result of the beef to pork redistribution. The values in the fourth column and the last two rows represent the GHG emission changes from the redistribution from eastern and western Canada, respectively, prior to the scenario assessment. The first three quantities in the last two lines show that the beef to pork redistribution resulted in lower annual emissions for all three GHGs, but especially for methane because of the ruminant digestion of forage by cattle.

**Table 4:** Comparison of the annual GHG emission budgets of the 2001 Canadian beef and pork industries before and after land redistribution due to increased pork production.

	TgCO_2_e				
Farm type	Region	CH_4_	N_2_O	CO_2_	GHGs
	Baseline annual GHG emissions prior to land redistribution				
Beef	East	2.64	2.06	0.49	5.19
	West	14.71	8.32	2.78	25.81
Pork	East	1.62	1.44	0.92	3.99
	West	1.46	0.77	0.83	3.06
	Deducted GHG emissions resulting from reduced beef production				
Beef	East	0.26	0.21	0.05	0.52
	West	1.47	0.83	0.28	2.58
	Additional GHG emissions resulting from increased pork production				
Pork	East	0.04	0.03	0.02	0.09
	West	0.26	0.14	0.15	0.54
	Net annual GHG emissions deducted from land redistribution				
	East	0.23	0.17	0.03	0.43
	West	1.21	0.69	0.13	2.04

**Table 5:** Annual GHG emissions from the residual forage area (∆*_r_*A) under four land use scenarios ^1^ in eastern and western Canada in 2001.

Scenario #	1	2	3	4
	TgCO_2_e			
East	0.00	0.19	0.40	0.50
West	0.00	0.29	1.48	1.81
Canada	0.00	0.48	1.88	2.31

^1^ four scenarios for ∆rA: Scenario 1: remains under perennial forage cover, Scenario 2: seeded to annuals, Scenario 3: returned to beef production with mixed forage and grain diet, Scenario 4: returned to beef production with mostly forage diet.

The estimated annual GHG emissions from ∆*_r_*A under the four scenarios are shown in Table 5. These GHG emissions were subtracted from the GHG reductions in Table 4 to estimate the net changes in GHG emissions under each scenario. Due to enteric methane, the highest GHG emissions resulted from Scenario 4, followed by Scenario 3. The fertilizer N_2_O and fossil CO_2_ from farm field operations under Scenario 2 resulted in lower annual GHG emissions than from Scenarios 3 and 4. There were no GHG emissions from ∆*_r_*A under Scenario 1 since ∆*_r_*A is supposed to remain under perennial forage. 

The expected 40 year losses in soil carbon [40] as a result of the four scenarios are shown Table 6 (rows 1 to 3). The net changes in annual GHG emissions associated with each of the four scenarios are then presented (rows 4 to 6). In Scenario 1, the net annual reduction in GHG emissions from Table 4 were compared to the changes in soil carbon under ∆*_c_*A. For Scenarios 2, 3 and 4, the annual GHG emission changes had to be compared to changes in soil carbon under both ∆*_c_*A and ∆*_r_*A. The payback periods in years required for reductions in annual GHG emissions to equal the 40 year cumulative losses in the soil carbon stock were determined from the ratios of the 40 year soil carbon losses (rows 1 to 3) to the respective decreases in annual GHG emissions (rows 4 to 6). 

**Table 6:** Changes in soil carbon and annual GHG emissions due to beef to pork redistribution, and payback periods required for decreased GHG to compensate soil carbon losses, under four scenarios ^1^ for using residual land ^2^ in 2001.

Scenario #	1	2	3	4
	Soil carbon loss over 40 years (Tg CO_2_e)			
East	0.10	9.57	2.12	1.67
West	7.67	54.48	15.48	13.32
Canada	7.77	64.06	17.60	14.99
	Decrease ^3^ in annual GHG emissions (Tg CO_2_e)			
East	0.43	0.24	0.02	−0.08
West	2.04	1.75	0.56	0.23
Canada	2.46	2.02	0.59	0.15
		Payback period (years)		
East	0.2	40.1	92.7	-
West	3.8	31.2	27.6	57
Canada	3.2	31.9	29.9	96.9

^1^ scenarios include four uses of residual land for crop or beef production ^2^ residual land includes the area freed by displaced beef population ^3^ negative quantities represent an increase in annual emissions

The largest loss of soil carbon came from reseeding all of ∆*_r_*A to annual crops (Scenario 2, Table 6). The lowest loss in soil carbon results from leaving all of the residual land under perennial ground cover without repopulating ∆*_r_*A with beef cattle (Scenario 1). Repopulating ∆*_r_*A with just a category of beef that is highly forage dependent (Scenario 4) resulted in slightly less soil carbon loss than from repopulating ∆*_r_*A with beef cattle fed a mix of forage and grain (Scenario 3) in both eastern and western Canada. The net annual GHG emission reductions for Scenarios 3 and 4 are appreciably lower than the net annual emission reductions in Scenarios 1 and 2. The negative result for the eastern emission quantity for Scenario 4 indicates a net increase in annual GHG emissions. Because this scenario resulted in a loss of GHG mitigation potential in the east, there was no need to relate this annual loss to the loss of soil carbon. 

Due to high methane emissions, payback periods were the highest for Scenario 4, even though the predominantly forage based cattle produced the second lowest decrease in soil carbon. The payback period for reseeding all residual perennial forage to annual crops (Scenario 2) was 13% longer than the mixed forage and grain fed beef herd (Scenario 3) in western Canada, but was only 40% as long as in the east. Due to the increase in annual GHG emissions in the east under Scenario 4, no payback period was shown for this case. For Scenario 3 in the east, the payback period was 2.3 times the 40 year benchmark period. In western Canada, the two ratios of payback to benchmark periods were 0.7 and 1.4 for Scenarios 3 and 4, respectively. For Scenario 2, the payback to benchmark period ratios were 1.0 and 0.8 for eastern and western Canada, respectively. For Scenario 1, the ratios were less than 0.1 for both eastern and western Canada. The very short payback periods for Scenario 1 were the result of the lowest change in soil carbon of any of the four Scenarios, as well as the unreduced GHG savings from the basic beef to pork redistribution. 

### 3.2. Discussion

From a purely GHG emissions perspective, the payback period of four years or less for Scenario 1 (Table 6) suggests that leaving ∆*_r_*A as unconsumed perennial growth was the best GHG mitigation option. While this benefit would require the elimination of livestock, Scenario 1 could be used to grow feedstock for cellulosic ethanol [46], or simply be set aside for environmental purposes like wildlife habitat. With payback periods at three quarters or less of the 40 year window, Scenario 2 represents a net gain in CO_2_ mitigation potential in the west. In the east, Scenario 2 was neutral with respect to the 40 year payback window. While Scenario 2 eliminated livestock, the annual crops in this scenario would increase global food supply. Scenario 3 in the west was the only case where repopulating ∆*_r_*A with beef cattle led to a net gain in GHG mitigation potential based on the payback period being less than 40 years. Scenario 3 also had a slight advantage over Scenario 2 in the west. This was because beef production conserves soil carbon stock and it is a lower input system than field crops. Due to the heavy dependence on silage corn, this GHG mitigation benefit was lost in the eastern beef industry under Scenario 3. Scenario 4 was the least promising mitigation option, in spite of lower losses of soil carbon than under Scenario 3. This was particularly true in the east where the net annual GHG emissions actually increased. 

The need to apply four scenarios to the basic beef to pork redistribution is consistent with previous studies involving the interaction between the beef industry and biofuel feedstock production [5,46]. Like the interaction with pork production in this study, these previous studies showed that displacement of beef with several types of biofuel feedstock has a range of outcomes depending on how the operators of the displaced beef farms respond. Similarly, whether or not the residual land in this analysis will be used sustainably depends largely on how much the beef farm operators will be allowed to share in the economic benefits of the land use shifts [46].

Because the beef to pork redistribution required land to be shifted from forage to annual grain crops, it was assumed that a sufficient portion (up to 10%) of the land under forage would be suitable for growing feed grains. Scenario 2 required that all of ∆*_r_*A be suitable for either cereal or pulse crop production. The main uncertainty in the beef to pork redistribution was the use of ∆*_r_*A. This was because that land may be used for either perennial or annual crops, or to support either food or biofuel feedstock production. It might also be allowed to revert back to rangeland or natural habitat, depending on the economic pressures. Hence, the four scenarios represent only a few of the many possible ways that livestock industries and soil carbon stocks can interact. When the GHG emissions from the Canadian livestock industries were assessed individually, the LCC approach allowed them to be treated as closed systems. However, once farm type interactions are introduced, inter-commodity land use changes can no longer be viewed in complete isolation because the different intensities of land do not result in equal exchanges of land areas. 

Exploring scenarios for the redistribution shifted the focus from direct impacts on ∆*_c_*A to indirect impacts on ∆*_r_*A. However, it was not the objective of this paper to determine whether or not repopulating ∆*_r_*A with beef was necessarily the best use of the residual crop land. Because these scenarios were only applicable to ∆*_r_*A, this assessment does not describe the carbon footprint of the entire production system associated with each of the four scenarios. For example, the annual field crops in Scenario 2 by themselves, do not replace the protein that could have been produced if either Scenario 3 or 4 had been the chosen land use option. Even though these last two scenarios have not been found to mitigate GHG emissions, both of them resulted in higher beef protein production. Also, there are recognized ancillary environmental benefits to beef production that enhance sustainability [43], but this broader perspective on the overall sustainability of this production system was beyond the scope of this paper. Therefore, this scenario assessment should be treated as a demonstration of how the annual GHG emission budgets and soil carbon interact under changes in land use.

## 4. Conclusions

The model presented in this paper, was developed in order to quantify the impacts of changes in annual GHG emissions and soil carbon on the carbon footprint of the Canadian livestock industry. The paper also presents a method of reallocating farm animals or land from ruminant to non-ruminant production systems. With the inclusion of changes in soil carbon storage, the ULICEES model can provide a more comprehensive comparison of the negative impact of enteric methane emissions with the benefit of protecting soil carbon under permanent cover. Similarly, other livestock commodities or non-enteric related GHG emission issues, such as manure management systems or tillage practices, can also be compared with changes in soil carbon with ULICEES. With the soil carbon interface, ULICEES can provide a more comprehensive comparison of GHG emission intensities of protein production among Canadian livestock types. 

Although it introduces an additional parameter to the GHG mitigation policy dialogue, the payback period approach was shown to be a potentially valuable indicator. For example, whereas reseeding all of the residual areas from displaced beef to annuals released the largest amounts of soil carbon, the savings in annual GHG emissions showed that this option could be a positive GHG emissions mitigation strategy over 40 years. However, given the vast reserve of grassland in western Canada that cannot support grain production, the lower carbon footprint of non-ruminants cannot override the fact that this land is only suitable for forage based beef production. 

This study has illustrated the complex interactions between livestock production industries that must be considered when attempting to balance agricultural production, land use and mitigation strategies. ULICEES shows promise as being an effective modeling tool for a wide range of land use and GHG mitigation policies in Canada. The results of the scenarios assessed in this paper suggest that conserving soil carbon stock did not compensate for the annual GHG emissions from forage based beef production in much of Canada. These findings should not, however, be interpreted as an indication that all farmland should be converted from beef to pork production. Assessments similar to this Canadian analysis could be done in other countries. However, in countries whose lack of food security would not justify grain based livestock production, or where most of the land resources are only suited to perennial forage production, ruminant livestock would continue to be the most sustainable, and often the only viable, food production system, regardless of the carbon footprint of that system.
